# Clinical aspects of cutaneous lupus erythematosus

**DOI:** 10.3389/fmed.2022.984229

**Published:** 2023-01-09

**Authors:** Julia Elmgren, Filippa Nyberg

**Affiliations:** ^1^Department of Medicine, Karolinska Institutet, Stockholm, Sweden; ^2^Department of Dermatology, Karolinska University Hospital, Stockholm, Sweden

**Keywords:** cutaneous lupus erythematosus, histopathology, classification of CLE, skin inflammation, lupus

## Abstract

Lupus erythematosus (LE) is an autoimmune inflammatory disease with a wide clinical spectrum from life-threatening multi-organ inflammation in systemic lupus erythematosus (SLE) to limited skin disease in cutaneous LE (CLE). The etiology of CLE is still not fully understood but a multifactorial genesis with genetic predisposition and certain environmental factors as triggers for the development are generally accepted features. Lesions can be induced and aggravated by UV-irradiation and smoking is linked to more severe forms of skin disease and to co-morbidity. Drugs, including many common medicines like antihypertensives, are known to induce subacute CLE (SCLE). The mechanisms involved have recently been shown to be part of the IFN-I pathway and new, specific treatments are currently in clinical trials. CLE is currently classified in subtypes based on clinical presentation and duration into acute CLE (ACLE), SCLE, and chronic CLE (CCLE). Distinct subtypes can be seen in individual patients or coexist within the same patient. Because of the confluent and overlapping picture between these subsets, serology, and histopathology constitute an important role guiding towards correct diagnose and there is ongoing work to update the classification. The Cutaneous Lupus Area Severity Index (CLASI) is a validated tool to measure activity and damage both in clinical trials but also for the clinician to evaluate treatment and follow the course of the disease among patients. CLE is known to have substantial impact on the life of those affected. Several tools have been proposed to measure QoL in these patients, currently Skindex-29 is probably the most used. Patient education is an important part of prevention of flares, including UV-protection and smoking cessation. First-line treatment includes topical corticosteroids as well as topical calcineurin inhibitors with the addition of systemic treatment with antimalarials in more severe or therapy resistant cases. Treatment specifically targeting CLE has been lacking, however novel potential therapies are in later phase clinical trials. In this review we aim to describe the different subsets of the cutaneous form in LE with focus on clinical aspects.

## Introduction

Descriptions of lupus (= wolf in Latin) can be found as early as the Middle Age. The first to describe Lupus Erythematosus in modern time was the Swiss dermatologist Laurent-Theodore Biett. In 1833 his work was published through his student Cazenave, giving it the name Erythema Centrifugum. Cazenave was among the first one to describe morphologically what today is known as discoid lupus (1). Two distinct forms of lupus were later described by Kaposi as erythematosus *discoides* and lupus erythematosus *disseminate*, which refer to a state of generalized lesions, i.e., manifestations below the neck (2).

## Classification of subtypes of CLE

In 1981 Gilliam and Sontheimer created a classification mostly based on clinical presentation of cutaneous characteristics in patients with lupus erythematosus (LE), and subdivided it into acute CLE (ACLE), subacute CLE (SCLE), and chronic CLE (CCLE) (3) Table 1. Different updates, additions and suggestions have been discussed widely. Other suggestions and additions such as the Düsseldorf classification proposed addition of a fourth type, named intermittent CLE (ICLE) (4). Further, suggestions of categorizing cutaneous LE (CLE) specifically based on histopathologic picture i.e., level of skin-involvement have also been proposed for classification (5). A Delphi process with international experts suggested 12 criteria for discoid LE (DLE), including morphology, histopathology and location, with ambition to reach homogeneity on the most common subset of CLE (6). These suggested criteria have been evaluated and found to probably be more applicable to disease damage than to evaluate disease activity. The clinical usefulness is still not clear, but these new criteria are considered to be of value when recruiting patients to clinical trials (7, 8). Management of CLE as well as clinical research is dependent on clear classification criteria and further work is needed to elucidate accurate and complete classification criteria.

**TABLE 1:** Classification of cutaneous LE suggested by Gilliam and Sontheimer (3) (modified) (3).

Chronic cutaneous LE
Clinical forms
1. Discoid LE (most common form)
^•^ Localized DLE
^•^ Generalized DLE (lesions above and below the neck)
2. LE profundus (panniculitis)
3. LE tumidus
4. Chilblain LE
5. Lichen planus overlap syndrome
Clinical features of DLE
Usually localized, chronic, scarring lesions of head and/or neck region lasting months to years. Usually no extracutaneous disease.
Subacute cutaneous LE (SCLE)
Clinical forms
1. Papulosquamous (psoriasiform)
2. Annular-polycyclic
Clinical features
Usually widespread, non-scarring lesions with associated scaling, depigmentation, and telangiectasis distributed on photo-sensitive areas.
Acute cutaneous LE (ACLE)
Clinical forms
1. Localized, indurated erythematous lesions (malar areas of face-“butterfly rash”)
2. Widespread indurated erythema (face, scalp, neck, upper chest, shoulders, extensor arms, and backs of hands)
Clinical and laboratory features
Multisystem disease and antinuclear antibodies are usually present.

### Epidemiology

Investigations from different parts of the world have shown CLE to have similar incidence figures as SLE. The global incidence of SLE is approximated as 1.5–11/100,000 per person-year, and in Europe 1.5–7.4/100,000 per person-year (9). The majority of those diagnosed with SLE are females with onset of disease in their third or fourth decade of life with a prevalence of 203/100,000 (10, 11).

Several epidemiological studies have been performed to determine the incidence and prevalence of CLE. In a study by Grönhagen et al. the population-based incidence of CLE in Sweden was found to be 4/100,000 per person years (12). Similar incidence rates have been reported from the US, Asia, and Denmark with a range of 2.74–4.36/100,000 per person-year (13–15). The ratio between biologic sex is overrepresented among females with a ratio of 2–4:1 (12, 14, 15). CCLE is overrepresented among racial/ethnic minority groups, particularly individuals with skin of color (16–18).

Discoid LEis the most common clinical presentation and is generally estimated to 62–83% of all CLE patients (12–15, 19). Also, DLE and SCLE are currently the only subtypes with specific ICD codes (L.93.0 and L93.1) while all other are classified as L93.2 “other localized cutaneous LE” making registry studies on a large scale impossible as tools to identify other, more rare subtypes.

A recent study found that signs of disease damage, particularly ear dyspigmentation, scalp dyspigmentation and scarring alopecia, can more frequently affect patients with skin of color with DLE (16).

### Association to SLE

Many shared features point to regarding CLE and SLE as being part of a disease spectrum: shared histopathological, clinical, and serological features as well as the presence of overlap and development from cutaneous to systemic disease. The risk for progression to SLE in DLE patients is estimated between 5 and 30%. The generalized form of DLE has a higher potential of progressing to SLE compared to those with localized lesions (12, 19–22). Potential risk factors for progression to systemic disease are suggested to be anemia, arthritis and positivity for ANA (19). DLE patients statistically have a lower risk of progression to or coexisting SLE compared to both ACLE and SCLE (23, 24).

However, there are also clinical differences to support regarding CLE as a distinct disease entity: underscoring this view is the low risk of DLE progressing to SLE and differences in genetic background, age and sex distribution.

Recently, lesional and serological B-cell expressions have been suggested to differentiate between cutaneous and systemic LE. B-cell activating factor (BAFF), a cytokine linked to activation of B-cells, seems to play an important role in SLE and has been elevated in 30% of patients. Increased expression of BAFF in lupus lesions compared to healthy controls has also been reported and theories suggest that levels of BAFF can correlate to disease activity (25). B-cell signature in lesions varied between the different subsets of CLE with the highest expression found in DLE lesions, suggesting that a gradient of expression can be identified (26, 27).

Of great interest for prognosis and management of CLE patients is to identify potential biomarkers. Biomarkers suggested to reflect a higher risk of progression from CLE to SLE are ANA, anti-ds- DNA-, anti- Sm-, anti-U1-RPN antibodies and higher erythrocyte sedimentation rate (ESR). For DLE, ANA positivity and anti-ds-DNA seem to be markers of risk for progression to SLE (28). Both SLE and CLE patients show elevations of IFN, therefore IFN-upregulation such as IFN-gamma may predict progression in SLE and could as well serve as a biomarker to predict progression among CLE patients (29). The IFN-regulated cytokine CXCL13 correlate with both disease activity in SLE and renal involvement. Widespread lesions are associated with a higher abundance of the ligand to CXCL13 and could therefore serve as a biomarker in the future (30). A suggested biomarker to evaluate response to treatment is vascular endothelial growth factor (VEGF) (31).

At the present state of knowledge, the two groups CLE and SLE are best regarded as closely related but distinct and different diseases. Correlation between SLE and CLE indicates that the overall risk of progressing to SLE is significantly higher within the first 3 years from CLE diagnosis (12, 13, 23). Studies from different parts of the world largely confirm the finding, that in case of systemic progression it occurs within a few years from cutaneous lupus diagnosis (14, 15). Epidemiologic findings therefore clearly underscore the importance of alertness for development of systemic disease, especially during the first years after diagnosis of CLE.

### Subtypes of CLE

#### Acute CLE

This subset occurs mostly in a patient with SLE and can be presented in a localized or a generalized form, with the former most recognized as an erythematous rash and edema over the malar eminences and bridge of the nose, although saving the nasolabial folds. This manifestation is usually triggered by UV irradiation although not exclusively. This so called “butterfly eruption” typically lasts from days to weeks and heals without scarring (24, 32). The rare, generalized form is presented as a morbilliform widespread eruption (24, 33). ACLE is often seen as a prodromal symptom of systemic disease and patients are usually positive for ANA (80%) and anti-dsDNA (30–40%) by this time (34). The lesions heal without scarring or dyspigmentation (20). Steven Johnson/Toxic Epidermal Necrolysis-like Lupus Erythematosus is a hyperacute manifestation of ACLE. It presents as a widespread erythema with epidermal detachment (20, 35). Other manifestations appearing with ACLE are telangiectasias, oral ulcerations, poikiloderma, scales, and erosions (24, 32).

Bullous SLE (BSLE) is a rare form of ACLE that was recognized by Hall et al. who reported about patients with vesiculobullous eruption with unknown etiology to disease and with poor response to corticosteroids. They instead used Dapsone and achieved significant results with remission close to administration (36). BSLE is considered a rare form and is mostly affecting adults between their thirties and forties and like SLE, this form predominately affects women. Clinical features of this form are widespread non-scarring blistering arising on erythematous or normal skin mostly affecting areas such as neck, trunk, and extremities. Histopathologic examination shows neutrophilic and interpapillary micro abscesses with a picture resembling those seen in dermatitis herpetiformis. Presence of autoantibodies against collagen type-VII have also been reported from several studies (37–40).

#### Subacute CLE

In 1979 Gilliam and Sontheimer proposed that this entity should be considered a distinct subset of LE (41). This subset is mostly described in Caucasian females and lesions usually occur in UV-exposed area such as neck, chest, back and arms but are rarely seen in the face (33). The classic presentation of SCLE lesions usually comes with an erythematous papules or macules that later progress to and become annular-polycyclic lesions or-, less common, hyperkeratotic papulosquamous lesions in the rarer psoriasiform type (42). A majority (about 80% depending of sensitivity of method) express autoantibodies anti-Ro/SSA- and often also anti-La/SSB antibodies (30–40%) (43). The lesions usually heal without scarring although dyspigmentation occurs (24). Among SCLE patients, around 50% present with a mild form of SLE reporting myalgia and arthritis as common symptoms but in contrary to systemic disease, few of these have manifestations in kidneys or central nervous system (44, 45). In a recent study, prevalence of AMA-M2 antibodies among patients with SCLE had an increase in cholestatic liver enzymes, suggesting patients with newly diagnosed SCLE to be screened for AMA. If present, the authors to this study recommend avoidance of drugs with potential liver toxicity in order to prevent a progression to primary biliary cholangitis (46).

#### Drug-induced SCLE

Since the first description of SCLE induced by thiazides in 1985 by Reed et al. the association with numerous drugs and drug-induced SCLE (DI-SCLE) is now well described and new drugs are added. Recently, the mRNA-COVID-19 vaccine has been associated to both induction and exacerbation of SCLE (43, 47–51).

Drug-induced SCLE is estimated to constitute about one third of all the SCLE and over 100 drugs have been associated to subacute DI-SCLE (43, 52). Of importance for clinicians to be aware of this condition when seeing patients with SCLE for the first time since it is identical to idiopathic SCLE (53).

However, some differences between idiopathic SCLE and DI-SCLE have been reported: Age of onset has been suggested to be higher in DI-SCLE, with a mean of 60 years compared to SCLE with a peak around 40 years (54). Reports of unique findings in DI-SCLE suggest characteristics as lesions with bullous and erythema multiforme type, more widespread, older age of onset and findings in histopathology described as leukocytoclastic vasculitis. The serologic findings of anti-Ro/SSA- and anti-La/SSB antibodies in most cases do not seem to differ between idiopathic and drug-induced form (52, 54).

Existing criteria for drug-induced SLE proposed by Borcher et al. have been proposed for application also in DI-SCLE (52, 55):

– sufficient and continuous exposure to a specific drug
– at least one symptom compatible with CLE
– no history suggestive of CLE before starting with drug
– resolution of symptoms within weeks after discontinuation of putative offending agent.

More rarely drug-induced CCLE has been reported, with typical discoid lesions in photo distributed areas (54).

Although this strong association, the relationships and pathomechanisms are not fully understood. Time from exposure to a new drug to onset can vary from days to years but median latency is approximated to 6 weeks. Most of these cases resolve once discontinuation and patients mostly improve clinically within 1–3 months (56). Depending on drug type, the improvement seems to vary in time from discontinuation ranging from months to years. Drugs with strong association include terbinafine, anti-tumor necrosis factor alfa (TNF-alfa) -inhibitors, PPIs, and monoclonal antibodies (MAbs) (43, 54, 57, 58). For the drug-induced discoid form of CLE association with 5-FU and anti-TNF-alfa are described (52). Recently a systematic review, covering therapy with MAbs, reported incidence of DI-SCLE, where the most common indication for MAb-treatment was inflammatory arthritis 40%, advanced melanoma 12% and psoriasis/psoriatic arthritis 10% (57).

### Chronic CLE

#### Discoid LE

The majority of patients within the CCLE group, have the discoid form (DLE) which can be presented as localized and generalized lesions (12–15). DLE are often coin shaped, erythematous, hyperkeratotic chronic lesions leaving scar behind mostly localized to head and neck with a lasting of months to years (3). In a review by Walling et al. the natural course of a DLE lesion starts as a macule or papule with a well demarked line with scaling that later progress to become a discoid plaque (59). DLE plaques are most often indurated and this has been suggested to be a criterion for DLE, however it is difficult to evaluate in a homogenous way and is not included in the current evaluation tool Cutaneous Lupus Area Severity Index (CLASI) (60). Histopathology is the gold standard for diagnosis, in typical cases it shows a hyperkeratosis and follicular plugging and interface dermatitis and a perifollicular lymphocytic infiltrate. Changes in basal layer of epidermis include membrane thickening as well as a more profound inflammatory infiltrate compared to ACLE and SCLE (4, 61). However, it is considered very difficult to discriminate between subtypes by histopathology alone. The histopathological finding of follicular hyperkeratotic plugs is also the most common finding by dermoscopy in DLE lesions as well as absence of follicular openings. Non-scalp lesions displayed a slightly higher frequency of hyperkeratotic plugs and red dots at dermoscopy compared to scalp lesions (62).

#### LE profundus (panniculitis)

This rare form of CCLE occurs in <5% of CLE and more seldom in SLE (53). It is of great importance for clinicians to recognize and treat this form since lesions can progress quickly and heal with subcutaneous atrophy scarring and dyspigmentation. An area affected by panniculitis often presents as a depression in the skin seemingly unaffected skin with palpable subepidermal nodules. The lesions can also present with a DLE plaque and erosion in the overlying skin. Lesions is usually located to proximal extremities, trunk and face but less commonly found in distal extremities (63). Histopathological changes in LE profundus show characteristics of panniculitis with mucin but there is no consensus in specific biopsy findings for LE profundus (64).

#### LE tumidus

This uncommon form of CLE was first reported in 1909 by dermatologist Erich Hoffman. This subtype is characterized by photosensitivity and “succulent” edematous erythematous plaques that heal without scarring, often in the face and more often prevalent in male patients than other forms of CLE. Locations that are most commonly affected is face, V-neck and back. A diagnose of LE tumidus is supported by histopathological findings of mucin and a lymphocytic infiltrate. Treatment is similar to other forms of localized CLE. Since 2012 LET is included in the SLICC as other forms of chronic CLE (65, 66).

#### Chilblain LE

This more rare subtype can occur both with and without SLE. It is most commonly found on the toes and fingers of females but can sometimes be more widespread. A history of cold-induced or aggravated lesions should be obtained (24). Patients are often anti-Ro/SSA antibody positive and some patients also display cryoglobulins at serological analysis (67). They also often have concomitant Raynaud’s phenomenon and are smokers. The lesions are tender, bright red to reddish-blue papules, nodules or plaques (24, 68).

Familial chilblain LE is a rare presentation caused by heterozygous mutations in the genes encoding 3‘repair endonuclease (TREX1) or corresponding protein. Familial chilblain LE typically begins in early childhood, and is associated with increased risk for SLE (69).

Chilblain Lupus is an example of a subset that has been linked to mutations in the genes *TREX1* and *STING*, i.e., a mutation in these regions will result in an IFN-1 immune response and disease activity (23, 70, 71).

#### LE-lichen planus overlap syndrome

This rare variant has clinical, histopathologic and immunofluorescence finding of both LE and LP (72).

### Neonatal LE (NLE)

Neonatal LE is a condition affecting offspring when maternal anti-Ro/SSA- and anti-La/SSB antibodies, and less common anti-U1 ribonucleoprotein is passed over placenta with the potential of inducing an inflammatory response (73). The exposure to antibodies is associated with an increased risk of autoimmune congenital heart block, skin rash, thrombocytopenia, leukopenia, anemia, and hepatobiliary disease. In concordance with theories of etiology to other autoimmune mediated diseases, NLE is a multifactorial disease with an interplay of genetic susceptibility within the child and the environment (73, 74). The skin manifestation associated with NLE is characterized of an erythematous rash with central clearing sometimes with scaling, resembling those seen in lesions of SCLE (75). It is typically located in periorbital area of the eyes, sometimes referred to as racoon sign. A histological examination would mainly show an interface dermatitis and accumulation of IgG in dermal-epidermal junction (DEJ). The lesions can be distributed in the face with a majority of 80%, but is also found in the scalp, trunk, and extremities. It is usually not present by time of delivery instead appearing weeks later lasting for months (75–77). The lesions may heal with hypopigmentation and telangiectasia but rarely leaving scars behind (75). The skin manifestation itself is harmless and with good prognosis of disappearing in correlation with clearance of antibodies. However, the more severe outcome associated with NLE are autoimmune mediated congenital heart block. This is, when occurring, an irreversible state and therefore require intervention with pacemaker (76). Among 0.20–0.86% of females are thought to be positive for anti-Ro/SSA antibodies, although a great number of these do not have manifestations and are therefore not aware of their positive serology/expression (78). In a prospective study performed by Jaeggi et al. they noted that high titers of anti-Ro/SSA- and anti-La/SSB antibodies correlated with an increased incidence of NLE in the child, thereby implicating that the levels of antibody titers exposure correlate with severity of symptoms in the child (74). Considering NLE a rare disease, a high number of women are not aware of their positive serology and approximately 1–2% of those with positive serology will give birth to a child with NLE therefore screening for antibodies is a topic that have been up to discussion among clinicians (79). Today diagnose is based on serology of the mother and clinical presentation in the offspring. According to practical guidelines, potential prevention tools are discussed such as prenatally treatment of mother with hydroxychloroquine (HCQ) and immunoglobulin. Pregnant women diagnosed with SLE are recommended to continue with HCQ preconceptionally and throughout their pregnancy (73, 80, 81).

For images of presented subtypes, we are referring to Rook’s textbook of Dermatology (82).

## Cutaneous manifestations in SLE and classification criteria

Classification criteria for SLE were developed in 1982 by the American College of Rheumatology (ACR). SLE diagnosis was initially based upon fulfillment of ≥4/11 criteria. The ACR criteria allow a patient with mainly mucocutaneous features of the disease, to fulfill 4 criteria (e.g., photosensitivity, malar rash, mucous ulcers, cutaneous lupus and ANA) (83). In 2012 the criteria were revised by The Systemic Lupus International Collaboration Clinic (SLICC) and were extended with additional 6 criteria. The 17 SLICC criteria gave a higher sensitivity in particular in an early phase of disease, although not higher specificity (84). In 2019 the EULAR/ACR criteria were further revised with adding a positive ANA test as an entry criterion, although of importance for clinicians to know that a negative test cannot exclude an SLE diagnosis (85). SLICC and EULAR/ACR both share high sensitivity (85–87).

Cutaneous manifestations in SLE are often divided into specific and non-specific, referring to specific histopathological picture or not (23). The non-specific manifestations can be seen in other systemic inflammatory diseases as well and is therefore not considered to be pathognomonic for CLE (88) (Table 2).

**TABLE 2:** LE non-specific skin manifestations.

Raynaud’s phenomenon –
Cutaneous vasculitis
Non-scarring alopecia
Livedo reticularis
Digital manifestations
Photosensitivity

## Mucosal lesions in SLE and CLE

In the EULAR/ACR classification criteria for SLE diagnosis, mucocutaneous lesion include oral ulcers as an additive criterion for an SLE diagnose (85). Mucosal lesions in lupus have been described with a variety of descriptive terms with no unified terminology. The prevalence of oral mucosal lesions and the various morphological presentations with possible correlation to disease activity was recently described and underscore the clinical importance of mucosal lesions also in a dermatological setting (89).

## Diagnosis and management of CLE

The diagnosis is based on clinical evaluation and confirmed with histopathological investigation of skin biopsy. Serology is routinely obtained at baseline to assess systemic involvement as well as guidance among the subsets of CLE.

### Dermapathology/histopathology

Histopathological picture for diagnosing CLE is considered the golden standard combined with clinical and serological picture. A biopsy will not be able to confidently discriminate between the three main subsets of CLE since these will all show an interface dermatitis. In the subsets of the less common forms of chronic CLE, especially tumidus and panniculitis, different histopathological features especially presence of mucin, have been widely discussed but consensus in criteria exist as of today (20). Moreover, the histopathological picture in cutaneous lesions of dermatomyositis, is identical to the picture seen in CLE (90).

### Immunofluorescence

The lupus band test (LBT) is not routinely performed in CLE, but it can be helpful in the differential diagnosis of different inflammatory conditions in the skin. Non-lesion LBT is recommended as a diagnostic adjunct for diagnosing SLE in inconclusive cases (91). LBT consists of Immunoglobulins, predominantly IgG but also IgM and IgA together with complement factors C1q and CR in a linear pattern at the dermal-epidermal junction shown by immunofluorescence techniques on skin biopsies. They are reported to occur in lesional and sunexposed skin in DLE and SLE in more than 80% of cases. A positive LBT in non-exposed (e.g., gluteal) skin is seen in approximately 50% of SLE patients, but when it is found it is regarded as a specific criterion for SLE (59, 92, 93).

### Serology

A serological test of ANA and extractable nuclear antigens (ENAs) should be performed at baseline to assess possible systemic involvement. A routine blood and biochemistry test including urinanalysis for proteinuria should be performed. If antimalarials are considered, a visual check should also be performed before start of medication.

ANA positivity is commonly present in ACLE together with anti-ds-DNA, but in less than 50% of DLE. Anti-Sm- as well as anti-ds-DNA positivity is not commonly present in DLE or SCLE but occur more frequently in ACLE (8).

### Differential diagnosis

Dermatomyositis (DM) is an important differential diagnosis, and in cases without or with minimal myositis can be very similar to SCLE both clinically and histopathologically (94). In a recently published study, proteomic analyses were conducted through skin biopsies with lesions both from DM and CLE. Findings in this study was expression of IL-16, which was highly abundant and detectable in CLE lesions while in DM not detectable. Interpretation of this novel finding into the clinic could assist clinicians to differentiate between DM and CLE since the histopathologic appearance is similar in these two entities (95).

Sjögren’s syndrome (SS), is primarily associated with Sicca symptoms, dry eyes and mouth, caused of an autoimmune reaction to lacrimal and salivary glands (96). Patients diagnosed with SS are frequently positive for anti-Ro/SSA-antibodies and sometimes SCLE and SS is seen in the same patient. It is, however not known why some patients with anti-Ro/SSA antibodies have increased frequency of photosensitivity and SCLE, and some are not photosensitive and display SS. The so-called annular erythema of SS is sometimes considered to be the Asian counterpart of SCLE, but there is no consensus in criteria or possible differences (97, 98).

## Quality of Life, general symptoms, and comorbidity

It is today well known that patients with various skin disorders experience a great burden on their mental wellbeing (99). Consequently, a diagnose with CLE will have impact both on the physical appearance and on the mental health. The prevalence of depression among SLE patients is higher compared to the general population and studies on patients with CLE also implicate that mental illness as well as depression is increased (18, 100). Pruritus is a contributing factor to the impaired QoL in a variety of skin conditions and systemic disorders including autoimmune connective tissue diseases. Studies have shown that pruritus is a common subjective symptom in CLE and even appeared to be comparable to the itch experienced in chronic idiopathic urticaria and late-stage T-cell lymphoma. Pruritus may be an underrecognized symptom in CLE and may be a marker for disease activity both in CLE and SLE (101). Regarding this fact, early diagnosis, adequate treatment, and close collaboration among clinicians underscore the importance to enable a holistic treatment (102). An increase of depressive symptoms has been found also in patients with DLE and skin of color although not correlated to disease-activity but rather due to socioeconomic factors (17, 18).

The question of an increased risk of cancer among CLE has been studied, although the incidence of cancer has been reported to be higher among CLE patient compared to the general population, studies have not been able to exclude potential confounders such as exogenous factors, e.g., smoking (103, 104). Patients with CLE also seem to have higher risk in diseases such as embolism and thrombosis (105).

## Guidelines of care

Current guidelines for management are based on clinical experience and consensus work as well Cochrane reviews (106–108). Before start of treatment, clinical assessment should be complemented by assessment of severity of activity and damage.

Cutaneous Lupus Area Severity Index was developed in 2005 as a tool for practicing clinicians to be used for measure damage and disease activity in cutaneous LE. Average time duration for assessment of CLASI is 5.25 min which makes it to a tool that is not time consuming and would therefore not interfere with time limit for appointment. Activity is defined as erythema (0-3p) and scale/hypertrophy (0-2p) and based on anatomic location. Damage is defined as scarring/atrophy/panniculitis (0-2p) and dyspigmentation (0-1p). Lesions in mucous membrane, alopecia and dyspigmentation are also part of scoring in CLASI (109). This tool has been validated against physician-reported and patient-reported outcomes in SLE (110). CLASI seems to contribute to a more comprehensive measurement as well as objective measure of the improvement in disease activity.

Ideally, patient reported measures of quality of life, using tools as DLQI, Skindex-29 and CLEQoL should also be checked at baseline before treatment and regularly followed up. At present there is no specific, standardized measure of QoL in CLE although some have been proposed (111–114).

Information including prophylactic measures such as smoking cessation and UV-avoidance are mandatory. A structured follow up of treatment results using CLASI as a tool, will help in guiding the clinician in the individual patient as well as creating knew knowledge and room for quality improvement work. Among CLE patients the prevalence of smoking is higher compared to the general population and according to Bartels et al. smoking exposure in pack years showed an increase in cutaneous manifestation among patients with SLE (115, 116). Smoking also seem to affect response to antimalarial agents resulting in worse response among patients whom receiving antimalarial treatment and smoke compared to non-smoking patients (117, 118). However, the mechanism is not fully understood, and important to be aware of smokers that among with CLE also tend to have more disease activity and therefore might be more challenging to monitor.

**FIGURE 1:**
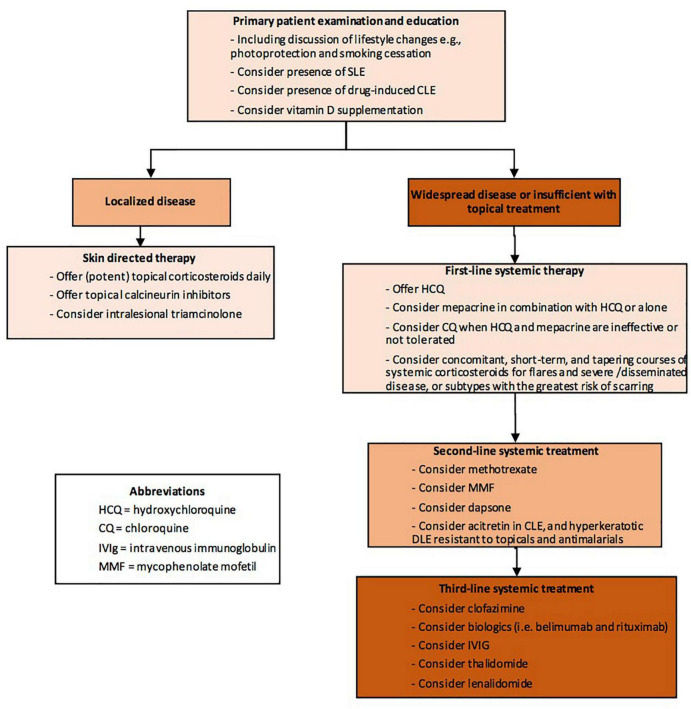
Patient management pathway Cutaneous Lupus Erythematosus (abbreviated) from Wiley Library © 2021 British Association of Dermatologists (119).

Currently, first line treatment is sunscreens, topical or intralesional corticosteroids or topical calcineurin inhibitors. In more widespread cases or if local treatment is not sufficient, antimalarial drugs are helpful in more than half of cases of CLE. Recently, a practical algorithm was published as a part of British Association of Dermatologists guidelines—“Patient management pathway” (Figure 1) (119).

Although hydroxychloroquine is regarded to be very safe concerning potential retinal toxicity, recent data suggest that a longer treatment time than 7 years should be monitored (120).

Patients not responding to antimalarials, may respond to other immunomodulatory agents such as oral corticosteroids, retinoids, dapsone, methotrexate, mycofenolate mofetil (MMF), acitretin, clofazimine, biologics, intravenous immunoglobulin (IVIg), thalidomide and lenalidomide (59, 119).

Clinical trials on specific treatment aimed at the recent knowledge of immunopathogenesis in the IFN-I pathway and different ways to block interferon production and effects, are ongoing (121–123). Recently, the use of anti-BDCA2 antibody Litifilimab in CLE patients in a phase-two study was reported to be superior to placebo. In this study the treatment target used was CLASI-activity score (124).

The therapy strategy treat-to-target (T2T) has gained recognition as an efficient therapeutic strategy for management of chronic diseases in terms of both medical outcome and patient satisfaction. The aim is to achieve remission or the absence of symptoms by identifying a treatment target followed by frequent controls and, if needed, modifications of therapy. This requires validated scoring systems to evaluate therapy outcome. SLE has been proposed as a condition with potential for the T2T strategy with promising results (125, 126). In CLE, structured use of T2T would require further validation of present tools for long-term disease outcomes such as CLASI and QoL instruments.

## Conclusion

Strict, accepted, and meaningful classification and treatment targets along with efficient new treatments will eventually lead to better outcomes for this patient group.

## Author contributions

JE performed literature search and writing. FN edited and commented and wrote parts of the text. Both authors contributed to the article and approved the submitted version.
